# Single-Nucleotide Variants in the AIM2 – Absent in Melanoma 2 Gene (rs1103577) Associated With Protection for Tuberculosis

**DOI:** 10.3389/fimmu.2021.604975

**Published:** 2021-04-01

**Authors:** Mariana Brasil de Andrade Figueira, Dhêmerson Souza de Lima, Antonio Luiz Boechat, Milton Gomes do Nascimento Filho, Irineide Assumpção Antunes, Joycenéa da Silva Matsuda, Thaís Rodrigues de Albuquerque Ribeiro, Luana Sousa Felix, Ariane Senna Fonseca Gonçalves, Allyson Guimarães da Costa, Rajendranath Ramasawmy, Alessandra Pontillo, Mauricio Morishi Ogusku, Aya Sadahiro

**Affiliations:** ^1^ Laboratório de Imunologia Molecular, Departamento de Parasitologia, Universidade Federal do Amazonas (UFAM), Manaus, Brazil; ^2^ Programa de Pós-Graduação em Imunologia Básica e Aplicada, Universidade Federal do Amazonas, Manaus, Brazil; ^3^ Laboratório de Imunogenética, Departamento de Imunologia, Instituto de Ciências Biomédicas (ICB), Universidade de São Paulo (USP), São Paulo, Brazil; ^4^ Policlínica Cardoso Fontes – Secretaria de Estado da Saúde do Amazonas-SUSAM, Manaus, Brazil; ^5^ Diretoria de Ensino e Pesquisa, Fundação Hospitalar de Hematologia e Hemoterapia do Amazonas (HEMOAM), Manaus, Brazil; ^6^ Instituto de Pesquisa Clínica Carlos Borborema, Fundação de Medicina Tropical Dr. Heitor Vieira Dourado, Manaus, Brazil; ^7^ Faculdade de Medicina Nilton Lins, Universidade Nilton Lins, Manaus, Brazil; ^8^ Laboratório de Micobacteriologia, Instituto Nacional de Pesquisas da Amazônia (INPA), Manaus, Brazil

**Keywords:** tuberculosis, inflammasome, SNV, *AIM2*, *CARD8*, *CTSB*

## Abstract

Tuberculosis (TB) remains a serious public health burden worldwide. TB is an infectious disease caused by the *Mycobacterium tuberculosis* Complex. Innate immune response is critical for controlling mycobacterial infection. NOD-like receptor pyrin domain containing 3/ absent in melanoma 2 (NLRP3/AIM2) inflammasomes are suggested to play an important role in TB. NLRP3/AIM2 mediate the release of pro-inflammatory cytokines IL-1β and IL-18 to control *M. tuberculosis* infection. Variants of genes involved in inflammasomes may contribute to elucidation of host immune responses to TB infection. The present study evaluated single-nucleotide variants (SNVs) in inflammasome genes *AIM2* (rs1103577), *CARD8* (rs2009373), and *CTSB* (rs1692816) in 401 patients with pulmonary TB (PTB), 133 patients with extrapulmonary TB (EPTB), and 366 healthy control (HC) subjects with no history of TB residing in the Amazonas state. Quantitative Real Time PCR was performed for allelic discrimination. The SNV of *AIM2* (rs1103577) is associated with protection for PTB (*p*adj: 0.033, ORadj: 0.69, 95% CI: 0.49-0.97). *CTSB* (rs1692816) is associated with reduced risk for EPTB when compared with PTB (*p*adj: 0.034, ORadj: 0.50, 95% CI: 0.27-0.94). Serum IL-1β concentrations were higher in patients with PTB than those in HCs (*p* = 0,0003). The SNV rs1103577 of *AIM2* appeared to influence IL-1β release. In a dominant model, individuals with the CC genotype (mean 3.78 ± SD 0.81) appeared to have a higher level of IL-1β compared to carriers of the T allele (mean 3.45 ± SD 0.84) among the patients with PTB (*p* = 0,0040). We found that SNVs of *AIM2* and *CTSB* were associated with TB, and the mechanisms involved in this process require further study.

## Introduction

Tuberculosis (TB) is an infectious disease caused by mycobacteria belonging to the *Mycobacterium tuberculosis* complex (MTBC) (1). The MTBC typically affects the lungs (pulmonary TB (PTB) but can also affect other tissues and organs (extrapulmonary TB (EPTB)) (2). Approximately 25% of the world’s population is infected with *M. tuberculosis*, but only approximately 5% to 10% of *M. tuberculosis*-infected individuals subsequently develop TB (3). The complexity of TB is influenced by several factors, such as the immune status and genetic factors of host (4–8).

The innate immune response is the first line of host defense against mycobacteria infection (9, 10). Innate immune system senses pathogens via pattern-recognition receptors (PRRs) to recognize conserved microbial components known as pathogen-associated molecular patterns (PAMPs). PRR also function as innate sensors of host-derived danger signals, the danger-associated molecular patterns (DAMPs) and may assemble in inflammasomes (11). Inflammasomes are intracellular multimeric protein complexes that play key roles in the innate immune response (12, 13). Inflammasomes are essential for controlling bacterial growth through the activation of caspase-1 to process pro-inflammatory IL-1β and IL-18 into active cytokines (14, 15). Caspase-1 also induces a type of inflammatory cell death called pyroptosis (16).

Inflammasomes belonging to the NOD-like receptor (NLR) family are composed of at least three components, a sensor protein (NLRP1, NLRP3, NLRP6, NLRP12, NLRC4; PYHIN family (PYD-like and HIN domain-containing proteins) as AIM2 and TRIM family (Tripartite motif proteins) containing PYRIN), an inflammatory caspase (caspase-1, Caspase-11) and an adapter molecule such as the apoptosis-associated speck-like containing a CARD domain (ASC) (17). NLRP3-inflammasomes is the most studied compared to others (18). NLRP3 and AIM2 inflammasomes play important role in host defense against *M. tuberculosis* (9, 15, 19–22).

The *NLRP3* gene is located on human chromosome 1. Basically, member of NLR family of PRR consists of three domains: a leucine-rich repeat (LRR)-region that interacts with antigens, a central nucleotide-binding NACHT domain and an effector domain PYD or CARD (23). *AIM2* gene is located on human chromosome 1. AIM2 is composed of two domains: a N-terminal pyrin domain (PYD) and a C-terminal hematopoietic interferon-inducible nuclear protein with a 200-amino acid repeat (HIN200) domain. AIM2 is a cytosolic double-stranded DNA receptor (24, 25).

Principles of inflammasome activation in TB involve two signals. The first signal induces the expression of NLRP3 and/or AIM2 inflammasome, pro-IL-1β and pro-IL-IL-18 genes caused by Toll-like receptor (TLR) stimulation. The second signal results to oligomerization domain and formation of inflammasome complex consequent of activating stimuli. After that, the effector domain recruits ASC protein to assemble the inflammasome complex (PRR, ASC protein and procaspase-1). ASC protein recruits procaspase-1 through caspase recruitment domain (CARD) consequently it is cleaved into active caspase-1 (14).

Activating stimuli described for NLRP3 are mitochondrial factors (mitochondrial reactive oxygen species, mitochondrial DNA, cardiolipin), mycobacterial components, cathepsins after lysosomal rupture as Cathepsin B encoded by *CTSB* gene located on human chromosome 8, potassium efflux and extracellular ATP via the P2X7 purinergic receptor (26, 27) and AIM2 is cytosolic dsDNA (25, 28). CTSB is cysteine exo/endopeptidase enzymes pH dependent belonging members of the papain family seems to involvement roles in NLRP3-inflammasome activation as well as degradation or processing of lysosomal proteins (29–31). CARD8 adaptor protein is encoded by *CARD8* gene present on chromosome 19. CARD8 acts as a negative regulator during NLRP3 inflammasome activation for adequate functioning and balanced response (32, 33).

In animal models of TB, NLRP3 (34, 35) and AIM2 (19, 36) inflammasomes have been found to recognize cytosolic DNA. Recently, the importance of inflammasomes in TB (37) as well as the crucial role of IL1β in infection control independent of inflammasome activation have been highlighted (38). TB is complex and multifactorial and identification of genetic variants can improve our understanding of the pathogenesis of TB (39).

Several studies have shown a genetic predisposition to development of TB (40–44). Genetic variant in inflammasomes can change their physiological function and contribute to the susceptibility, severity, and outcomes of TB (45). Single-nucleotide variant (SNV) of *NLRP3* (rs10754558) and SNV of *P2X7* (rs2230911) are associated with TB. SNV of *NLRP3* (rs10754558) is associated with protection against PTB (41). The SNV of *P2X7* (rs2230911) is associated with susceptibility to PTB. SNVs of *CARD8* (rs2043211) and *NLRP3* (rs35829419) are associated with EPTB in Ethiopian population (46).

The important role of inflammasomes in immunity to TB prompted us to evaluate the associations of SNVs in genes encoding the AIM2 inflammasome, CARD8, and CTSB in patients with TB from Amazonas state of Brazil.

## Material and Methods

### Ethics Statement

This study was approved by Human Research Ethics Committee of Federal University of Amazonas (N°. CAAE: 57978916.3.0000.5020; August 17, 2016). All patients and controls participating in this study provided written informed consent for collection of blood and sputum samples for analysis.

### Study Population

This is a case–control study and is consisted of 401 patients with PTB, 133 patients with EPTB, and 366 healthy controls (HCs). The study population was recruited from Policlínica Cardoso Fontes, a sanitary pneumology center (Manaus, AM, Brazil). The patients with PTB were positive for *M. tuberculosis*, which was determined through a molecular rapid test (TRM, GeneXpert MTB/RIF) (47) or through sputum smears and PKO culture method (47, 48). The diagnosis of EPTB was based on recommendations of Brazilian National Guidelines for the Control of Tuberculosis (49). The patients with EPTB were either sputum MTB-positive or culture-positive and were molecularly characterized for MTBC. The clinical manifestations of EPTB were pleural cutaneous, ganglionic, intestinal, perianal, ocular, bone, and miliary.

HCs participating in study had no history of TB and were contacts of patients with TB. The HCs were devoid of TB symptoms and negative for *M. tuberculosis* in sputum and/or in culture tests. Exclusion criteria comprised current pregnancy, recipients of organ transplants, and presence of other comorbidities, such as cancer, diabetes, HIV positive, hepatitis, and autoimmune diseases.

### DNA Isolation and SNV Genotyping

Genomic DNA was extracted from whole human blood (1 mL) using tetramethylammonium bromide salts protocol (50). SNVs were selected according to the minor allele frequency (MAF) of > to 10% in the global population from NCBI database, but also for background information on inflammasome complex in tuberculosis. Three SNVs in inflammasome genes *AIM2* T>C (rs1103577) situated on chromosome 1q15, *CARD8* T>C (rs2009373) on 19q48, and *CTSB* A> C (rs1692816) on 8q11 were selected and genotyped using TaqMan probes for allelic discrimination assay by Real-Time PCR System (Applied Biosystems). The technology is based on hydrolysis fluorescent probes. The fluorescent probes are allele-specific oligonucleotides sequences of target SNV labeled with either VIC or FAM fluorescence to discriminate the alleles. Thermal cycling conditions for PCR were a pre-read stage at 60^o^C for 30 sec and subsequently a hold stage at 95^o^C for 10 min followed by 50 cycles of denaturation at 95^o^C for 15 sec and annealing\extension at 60^o^C for 01 sec and finally followed by a post-read stage at 60^o^C for 30 sec. The reaction mix contained 5 μL TaqMan Genotyping Master Mix 1X; 0,5 μL TaqMan® probes 20X of target SNV, 4,5 μL Milli-Q water and 2 μL DNA (50ng\uL). For each PCR plate, a negative control and three known positive controls of target SNV (rare homozygote, wild-type homozygote and heterozygote) were included to increase the reliability of assay. Of note, the SNV rs35130877 of the *AIM2* gene was previously studied in the same population (41). This was carried out using QuantStudio™ 3 (ThermoFisher Scientific) and Design & Analysis Software v.1.4.2. The probes used in experiment are shown in **Supplementary Table 1**.

### Cytokine Measurements

Human IL-1β levels were measured in plasma sample from patients with PTB prior to start of drug therapy and HCs using a commercially available enzyme-linked immunosorbent assay (ELISA) kit (BioLegend ELISA MAX™ Deluxe Sets) following the manufacturer’s instructions. 50 μL of plasma samples were added to each well of high binding plate and readings were performed on a BioRad spectrophotometer plate as recommended by kit, using the 450 nm filter. Samples and standards were analyzed in duplicate. The minimal detectable concentration of IL-1β for this kit was 0.5 pg/mL.

### Statistical Analysis

For each SNVs analysis, alleles analysis was performed using on the link https://ihg.helmholtz-muenchen.de/ihg/snps.html and the associations between the allelic/genotype frequencies among patients with PTB, EPTB, and HCs were examined using the package “SNPassoc” version 1.9-2 (https://cran.r-project.org/web/packages/SNPassoc/index.html) for R software version 3.4.3 (www.r-project.org). The best genetic model was performed via Akaike information criterion (AIC). The Hardy-Weinberg equilibrium was evaluated for all SNVs. The results were shown as the odds ratio (OR) and 95% confidence intervals (95% CI) from multivariate logistic regression analyses. To control the potential confounding factors, adjusted OR (OR_adj_) values for age and sex were provided. The multivariate logistic regressions were performed using STATA 15 (StataCorp Texas, USA) after adjusting for age and sex. To compare multiple means of cytokine levels, one-way analysis of variance was applied with the Tukey’s post-hoc test adjusted for multiple comparisons. A *p*-value less than 0.05 was considered as statistically significant.

## Results 

### Characteristics of the Study Population

A total of 900 unrelated individuals born in the North of Brazil participated in this study. Of 900 individuals, 534 and 366 were patients with TB and HCs, respectively. Among those with TB, 401 had PTB and 133 had EPTB. The mean ages of patients with PTB, EPTB, and the HCs were 38.1 ± 13.7, 34.6 ± 14.1, and 32.9 ± 12.5, respectively. Of patients with PTB, EPTB, and HCs, 243 (60.6%), 75 (56.4%), and 180 (49.2%) were male, respectively. Patients with EPTB exhibited mostly pleural (55.6%), followed by cutaneous (17.3%) and ganglionic (16.5%). Intestinal, perianal, ocular, bone, and miliary TB accounted for 10.5%.

### Analysis of the SNVs in Inflammasome Genes

All SNVs studied *AIM2* rs1103577, *CARD8* rs2009373 and *CTSB* rs1692816 were in the Hardy-Weinberg equilibrium (HWE) in both HCs and patients with TB. The frequencies of alleles and genotypes as well as the different comparisons are shown in **Tables 1**–**4**. Genotypes and the best inheritance modeling for *AIM2*, *CARD8* and *CTSB* genes are reported in **Supplementary Table 2**.

**Table 1:** Genetic models of association SNV rs1103577 of *AIM2* gene adjusted for sex and age in patients with pulmonary tuberculosis (PTB) and healthy control.

AIM2 Genetic Model	PTB n, %	Controls n, %	p value _adj_	OR _adj_ (95% CI)	AIC
Codominant			0.083	1.00	988.4
CC CT TT	124 (0.32) 180 (0.47) 81 (0.21)	95 (0.27) 180 (0.51) 76 (0.22)		_ 0.68 (0.48-0.96) 0.72 (0.47-1.11)	
Dominant			**0.027**		986.5
CC CT-TT	124 (0.32) 261 (0.68)	95 (0.27) 256 (0.73)		_ 0.69 (0.50- 0.96)	
Recessive			0.672		991.2
				_ 0.92 (0.64-1.33)	
CT-CC TT	304 (0.79) 81 (0.21)	275 (0.78) 76 (0.22)			
Overdominant			0.096		988.6
CC-TT CT	205 (0.53) 180 (0.47)	171 (0.49) 180 (0.51)		_ 0.78 (0.58-1.05)	
Log-Additive 0,1,2	385 (0.52)	351 (0.48)	0.096	0.84 (0.68-1.03)	988.6

PTB (pulmonary tuberculosis); adjusted for sex and age (p value _adj_, OR_adj_); 95% confidence interval (CI); AIC, Akaike information criterion value.

The results statistically significant are highlight in bold characters.

**Table 2:** Genetic models of association SNV rs2009373 of *CARD8* gene adjusted for sex and age in patients with extrapulmonary tuberculosis (EPTB) and pulmonary tuberculosis (PTB).

CARD8 Genetic Model	EPTB n, %	PTB n, %	p value _adj_	OR _adj_ (95% CI)	AIC
Codominant			0.083	``1.00	462.3
				_ 0.98 (0.59-1.64) 0.48 (0.23-1.01)	
CC CT TT	31 (0.34) 50 (0.54) 11 (0.12)	111 (0.30) 183 (0.49) 82 (0.22)			
Dominant			0.444		464.6
CC CT-TT	31 (0.34) 61 (0.66)	111 (0.30) 265 (0.71)		_ 0.82 (0.50-1.35)	
Recessive			**0.026**		460.3
CT-CC TT	81 (0.88) 11 (0.12)	294 (0.78) 82 (0.22)		_ 0.48 (0.25-0.96)	
Overdominant			0.322		464.2
				_ 1.26 (0.80-2.00)	
CC-TT CT	42 (0.46) 50 (0.54)	193 (0.51) 183 (0.49)			
Log-Additive			0.084		462.2
				0.75 (0.53-1.04)	
0,1,2	92 (0.20)	376 (0.80)			

PTB (pulmonary tuberculosis); EPTB (extrapulmonary tuberculosis); adjusted for sex and age (p value _adj_, OR_adj_); 95% confidence interval (CI); AIC, Akaike information criterion value.

The results statistically significant are highlight in bold characters.

**Table 3:** Genetic models of association SNV rs1692816 of *CTSB* gene adjusted for sex and age in patients with extrapulmonary tuberculosis (EPTB) and healthy control subjects.

CTSB Genetic Model	EPTB n, %	Controls n, %	p value _adj_	OR _adj_ (95% CI)	AIC
Codominant			0.120	1.00	310.8
				_ 0.49 (0.24-1.00) 0.83 (0.39-1.73)	
AA AC CC	19 (0.37) 17 (0.33) 15 (0.29)	93 (0.27) 169 (0.48) 89 (0.25)			
Dominant			0.120		310.6
				_ 0.61 (0.33-1.13)	
AA AC-CC	19 (0.37) 32 (0.63)	93 (0.26) 258 (0.74)			
Recessive			0.545		312.7
AA-AC CC	36 (0.70) 15 (0.30)	262 (0.75) 89 (0.25)		- 1.22 (0.64-2.34)	
Overdominant			**0.046**		309.1
AA-CC AC	34 (0.67) 17 (0.33)	182 (0.52) 169 (0.48)		_ 0.54 (0.29-1.00)	
Log-Additive			0.543		312.7
0,1,2	51 (0.13)	351 (0.87)		0.88 (0.59-1.32)	

EPTB (extrapulmonary tuberculosis); adjusted for sex and age (p value _adj_, OR_adj_); 95% confidence interval (CI); AIC, Akaike information criterion value.

The results statistically significant are highlight in bold characters.

**Table 4:** Genetic models of association SNV rs1692816 of CTSB gene adjusted for sex and age in patients with extrapulmonary tuberculosis (EPTB) and pulmonary tuberculosis (PTB).

CTSB Genetic Model	EPTB n, %	PTB n, %	p value _adj_	OR _adj_ (95% CI)	AIC
Codominant			0.084	1.00	314.9
AA AC CC	19 (0.37) 17 (0.33) 15 (0.29)	103 (0.27) 193 (0.50) 89 (0.23)		_ 0.50 (0.25-1.01) 0.98 (0.47-2.07)	
Dominant			0.177		316.1
AA AC-CC	19 (0.37) 32 (0.63)	103 (0.27) 282 (0.73)		_ 0.65 (0.35-1.20)	
Recessive			0.269		316.7
AA-AC CC	36 (0.71) 15 (0.29)	296 (0.77) 89 (0.23)		_ 1.46 (0.76-2.80)	
Overdominant			**0.026**		312.9
				_ 0.50 (0.27-0.94)	
AA-CC AC	34 (0.67) 17 (0.33)	192 (0.50) 193 (0.50)			
Log-Additive			0.841		317.8
0,1,2	51 (0.12)	385 (0.88)		0.96 (0.64-1.45)	

PTB (pulmonary tuberculosis); EPTB (extrapulmonary tuberculosis); adjusted for sex and age (p value _adj_, OR_adj_); 95% confidence interval (CI); AIC, Akaike information criterion value.

The results statistically significant are highlight in bold characters.

The distribution of SNV of *AIM2* (rs1103577) genotypes is slightly different between patients with PTB compared with HC (*p*_adj_: 0.083). The frequency of genotype CC was slightly higher among patients with PTB (32%) than among HCs (27%). The SNV of *AIM2* (rs1103577) was associated with a reduced risk of developing PTB (*p*_adj_: 0.027, OR_adj_: 0.69, 95% CI: 0.50-0.96) in a dominant model (CC versus CT + TT) when compared with HCs (**Table 1**). Carriers of T allele had 31% less chances of developing PTB compared to individuals homozygous for C allele, suggesting homozygosity for C allele may be a risk factor for PTB.

The SNV of *CARD8* (rs2009373) showed a decrease risk in development of EPTB when compared to PTB in a recessive model (*p*_adj_: 0.026, OR_adj_: 0.48, 95% CI: 0.25-0.96) (**Table 2**). Bearers of C allele had 52% less chance of developing PTB. The frequency of genotype TT was predominant among the patients with PTB (22%) than in the EPTB group (12%). Individuals homozygous for T allele had twice chance of developing PTB than EPTB when compared with individuals homozygous for C allele (TT vs. CC *p* = 0.05, OR=2.1, 95% CI: 0.99-4.4) (The results of the different alleles were extracted on the link https://ihg.helmholtz-muenchen.de/ihg/snps.html). Of note, the frequencies of genotype were 31%, 49% and 20% for CC, CT and TT respectively, among HCs group (**Supplementary Table 2**). Comparisons between patients with EPTB and HCs revealed that carriers of C allele (CC +CT vs. TT) had 85% risk of developing EPTB (*p*=0.07, OR = 1.85, 95% CI: 0.94-3.7). No association was revealed when patients with PTB were compared with HCs.

Regarding the SNV of *CTSB* (rs1692816), an overdominant model indicated that heterozygous individuals have a lower risk of developing EPTB compared to patients with PTB (*p*: 0.022, OR: 0.50, 95% CI: 0.27-0.92) (**Supplementary Table 2**) and when adjusted for sex and age (*p*: 0.026, OR: 0.50, 95% CI: 0.27-0.94) (**Tables 3** and **4**). The frequencies were 23%, 50% and 27% for the CC, AC and AA genotypes, respectively among patients with PTB and similar pattern was observed among HCs (CC 25%, AC 48% and AA 27%). In contrast, the frequencies among patients with EPTB were 29%, 33%, and 37% for CC, AC and AA genotypes, respectively. Similarly, comparison between patients and HCs disclosed that heterozygous individuals have lower risk of developing EPTB (*p*: 0.045, *p*_adj_: 0.046, OR: 0.54, 95% CI: 0.29-1.0).

Logistic regression analysis, including sex and age variables with SNVs, confirmed the association of *AIM2* rs1103577 (*p*:0.033, OR: 0.69, 95% CI: 0.49-0.97) and *CTSB* rs1692816 with a lower risk for EPTB (*p*: 0.034, OR: 0.50, 95% CI: 0.27-0.94) (**Table 5**).

**Table 5:** Multivariate logistic regression analysis for variables sex and age, including three inflammasomes genes in the studied groups.

Variable	Tuberculosis vs Control		Pulmonary tuberculosis vs Control		Extrapulmonary tuberculosis vs Control		Pulmonary vs Extrapulmonary tuberculosis	
	*p* value	OR (95% CI)	*p* value	OR (95% CI)	*p* value	OR (95% CI)	*p* value	OR (95% CI)
*AIM2* rs1103577	0.053	0.73 (0.53-1.00)	**0.033**	0.69 (0.49-0.97)	0.241	0.68 (0.35-1.29)	0.976	1.00 (0.53-1.90)
*CARD8* rs2009373	0.825	0.95 (0.70-1.31)	0.652	0.92 (0.66-1.28)	0.539	1.21 (0.64-2.28)	0.279	1.41 (0.75-2.67)
*CTSB* rs1692816	0.229	0.83 (0.63-1.11)	0.597	1.08 (0.80-1.46)	0.066	0.55 (0.29-1.03)	**0.034**	0.50 (0.27-0.94)

odds ratio (OR) and 95% confidence interval (CI).

The results statistically significant are highlight in bold characters.

### Cytokine IL-1β in a Dominant Model of *AIM2*, *CARD8*, and *CTSB* Genotypes

**Figure 1:**
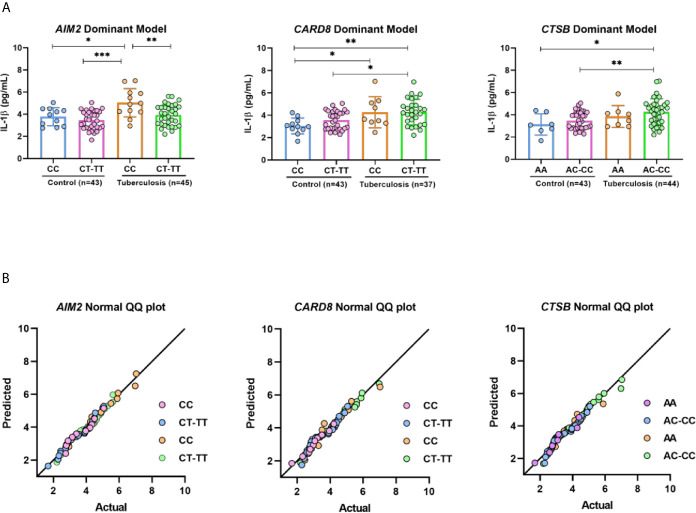
IL-1β profile respective on genotypes of *AIM2*, *CARD8* and *CTSB* from pulmonary tuberculosis patients and control. **(A)** Serum concentrations of IL-1β distributed in *AIM2*, *CARD8* and *CTSB* dominant model genotypes. **(B)** Graphic Normal QQ plot for three SNV studied. Groups were further compared with each other using ANOVA test with Tukey’s multiple comparisons test, *p < 0,05; **p < 0,001; ***p < 0,0001.

IL-1β is an important inflammatory cytokine with major role in host immune response against *M.tuberculosis* infection. Next, we evaluated *in vivo* the plasma IL-1β concentrations in PTB and HCs. Plasma IL-1β was higher in patients with PTB compared to those in HC group (*p* = 0.0003) (**Supplementary Figure 1**). We analyzed the distribution of plasma IL1-β according to genotypes of *AIM2*, *CARD8*, and *CTSB* in a dominant model. *AIM2* rs1103577 appeared to have an effect on plasma cytokine IL-1β when CC carriers were compared to allele T carriers (CC vs. CT+TT) in patients with PTB (*p* = 0.0040). CARD8 rs2009373 and CTSB rs1692816 did not show any influence on levels of plasma IL-β (**Figure 1A**). A QQ plot was used to validate the distribution of IL-1β in genotypes (**Figure 1B**).

## Discussion

Genetics and environmental factors are crucial for host immune response against *M. tuberculosis*. The complex network of mechanisms from early infection to development of TB remains poorly understood. Since the identification of function of various inflammasomes, several studies of genetic variants of genes involved in assembling the multiprotein complex of inflammasomes have attempted to determine the reason of susceptibility of some individuals to develop diseases while others remain asymptomatic. In the early stage of infection, innate immune response is essential to ensure the success of control and elimination of bacilli. Subsequently, upon inflammasomes activation, proinflammatory cytokines are released to keep in check the invading pathogen (45). In this context, we have focus on one SNV in the *AIM2* inflammasome in tuberculosis together with two other SNVs, one in *CARD8* and one in *CTSB*, that are related to NLRP3 pathway.

Studies regarding human genetic variants of *AIM2* are rare in infectious diseases (41, 42, 45). Recently, we investigated another SNV of *AIM2* (rs35130877) in 288 patients with PTB and 288 HCs. None of participants had this SNV (41). In this study, we found that SNV of *AIM2* (rs1103577) was associated with protection against TB. Interestingly, immunological studies involving mice have demonstrated a protective role of AIM2 against *M. tuberculosis* infection (19, 51). Inhibition of inflammasomes decreased the survival of *M. tuberculosis* in mice (37) and suggested that a high level of IL-1β favored the survival of bacteria. Carriers homozygous for C allele have higher IL-1 concentrations compared to T allele carriers, and T allele is associated with protection in our study. Furthermore, modern lineages of MTBC have high multiplication rates, which correlates to high levels of IL-1β (52). However, some individuals are resistant to *M. tuberculosis* infection despite continuous exposure (53, 54), suggesting sterile clearance potentially due to a reflex of genetic background and immunity of individual.

IL-1β is a proinflammatory cytokine and has been suggested to play key role in host protection against *M. tuberculosis* infection (55, 56). The protein ESAT of *M. tuberculosis* is cited as a potent activator of NLRP3\ASC inflammasome to liberate mature IL-1β (9). Patients with PTB also exhibit increased levels of IL-1βcompared to HCs. Patients with PTB also demonstrate higher levels of IL-1β (37, 38, 57), reinforcing an important role in the early innate immune response to control *M. tuberculosis* infection. We found that the serum concentrations of cytokine IL-1β were correlated according to genotypes CC versus CT-TT (*AIM2*) among patients with PTB in a dominant model. The T allele (TT +CT) correlated with low level of IL1 while homozygosity for the C allele with high level of IL-1β. The T allele is associated with protection to development of TB while individuals homozygous for C allele with susceptibility. Interestingly, excessive levels of IL-1β has been associated with severe TB and lung damage (57, 58).

NLRP3 and AIM2-inflammasome are suggested to play important role in host-defense against mycobacteria (19, 59). Recently, Souza de Lima et al. (22) have shown that NLRP3/IL-1ß/IL-18 pathway is strongly activated in a cathepsin-dependent form by virulent strain H37Rv and non-virulent BCG strains of *M. tuberculosis* in human macrophages, in vitro. Interestingly, the response was modulated according to macrophage donor genotype of SNV NLRP3 rs10754558 that correlated to level of IL-1β release (22). Of note, IL-1β is one among key proinflammatory cytokines essential for recruitment of immune cells to the site of *M. tuberculosis* infection (55). Several studies have demonstrated the role of AIM2-inflamasome in mycobacterial infection. Mice macrophages infected with pathogenic strain of *M. bovis* led to activation of AIM2-inflammasome and mature IL-1β release (60). Similarly, another study also demonstrated that THP-1 macrophages exposed to rBCG (Recombinant BCG ΔureC::hly) vaccine results in AIM2-inflammasome activation with increased production of IL-1β and IL-18 and autophagy, corroborating the role of AIM2-inflammasome in innate immune response (61).

AIM2-deficient mice infected with *Mycobacterium bovis* Bacillus Calmette-Guérin (BCG) showed a higher infection burden and developed severe disease due to simultaneous induction of reactive IFN-β and IFN-γ responses compared to wild-type mice (51). The AIM2 inflammasome appears to play a protective role through the induction of IL-1β and negative regulation of type I IFN induction. High production of type I IFNs reduces the IFN-γ response in TB infection (51). Interestingly, we showed that individuals homozygous for C allele had higher level of IL-1β compared to carriers of T allele and T allele is associated with protection to the development of TB. Higher level of IL-1β in the early stage might be important in keeping in check the pathogen but at a later stage might cause tissue damage.

The SNVs of *AIM2* (rs1103577), *CARD8* (rs2009373) and *CTSB* (rs1692816) are located in intronic region of respective gene. Although the functional role of these variants has not been elucidated, non-coding regions can affect gene expression resulting in different responses to presence of pathogens and can serve such as genetic markers (62). In future studies, it will be interesting to quantify the AIM2 messenger RNA (mRNA) from macrophages of different genotypes of *AIM2* (rs1103577) upon mycobacterial infection. Intronic regions have been highlighted for increasing the expression of mRNA in very diverse organisms (mammals, plants). Thus being an important regulator in the expression of proteins (63–66).

In this study, stratification of patients into PTB and EPTB suggested that *CARD8* rs2009373 and *CTSB* rs1692816 were associated with a lower risk of developing PTB. However, multivariate logistic regression analysis showed that only *CTSB* rs1692816 was associated with a reduced risk for EPTB. Indeed, we observed that heterozygosity for the variant provides lower risk for development of EPTB. CTSB is a member of cathepsin family localized in lysosomes and cytosol. CTSB promotes the degradation of protein in lysosomes and controls autophagy (67). Furthermore, the inhibition of CTSB blocks *M. tuberculosis*-induced NLRP3 inflammasome assembly, thereby leading to a decrease in IL-1βrelease, suggesting that the release of lysosomal CTSB and possibly other cathepsins are crucial for activation of NLRP3 to control *M. tuberculosis* infection (22, 30). Although, the function of this *CTSB* rs1692816 is not known, we can speculate that heterozygous individuals have an advantage in modulating the NLRP3 inflammasome in the release of IL-1β that is sufficient to keep in check the bacteria in comparison to homozygotes. Indeed, heterozygosity for some genes variant have been shown to provide an advantage to some infectious diseases. Heterozygosity for hemoglobin S is an advantage in Africa against *Plasmodium falciparum* malaria. Heterozygosity for the MAL/TIRAP variant in the TLRs pathway have been suggested to provide protection against invasive pneumococcal disease, malaria, tuberculosis and chagas disease (68, 69).

The SNV of *CARD8* (rs2009373) was not associated with TB in this study. However, other variants of *CARD8* (rs6509365 and rs2043211) have been associated with susceptibility to TB (41, 46, 70). CARD8 negatively regulates NLRP3 activation, and *CARD8* rs2043211 appears to have a loss of function. Interestingly, *NLRP3* (rs35829419)/*CARD8* (rs2043211) interaction is associated with levels of IL-1β (71).

Another important aspect is the variation in allele frequencies among populations. We found that some alleles were present in high or low frequencies or were entirely absent in specific populations, thereby indicating different evolutionary histories that are assumed are under selective pressure from prevalent diseases.

The distribution of MAF for rs1103577 of *AIM2* (T allele) in HCs (T = 0.47) is comparable to that of a European population (T = 0.40) and different from that of an African population (C = 0.09). The Amazonian population is an admixture of approximately 60% American, 50% European, and 10% African ancestry (72). The MAF of *CARD8* rs2009373 (T allele) in our group (T = 0.45) was similar to frequencies of South Asian (T = 0.46), European (T = 0.47), American (T = 0.41), and African (0.50) populations, but different from those of West Asian populations (C = 0.23). For rs1692816 of *CTSB*, both alleles A and C are frequent in different populations. The frequency of *CTSB* rs1692816 C allele was 0.49 in our population, compared to African (0.56), American (0.59), and European (0.66) populations. However, the frequency of the rs1692816 C allele was similar to that of East (0.50) and South (0.53) Asian populations. The frequencies of the different alleles were extracted from http://www.ensembl.org/. These frequencies indicate the importance of careful selection of cases and controls from same homogeneous population to avoid spurious associations.

The present study had some limitations. First, the sample size of the cases was small, especially for patients with EPTB. Second, we could not assay IL-1β in patients with EPTB due to a lack of biological samples. Additionally, the *NLRP3* data deviated from the HWE in both cases with TB and HCs; thus, they were excluded.

This result corroborates previous studies carried out by several researchers, both in an experimental model and in human cells infected by mycobacteria, which suggest the critical role of AIM2 in tuberculosis. We suggested *AIM2* rs1103577 was found to be associated with a lower risk of developing PTB and genotypes CC for AIM2 rs1103577 patients with PTB demonstrate higher levels of IL-1β. The *CARD8* and *CTSB* genes were also found to be important targets for TB, and other genetic variants should be investigated. Further studies are needed to confirm the association in other populations. Finally, it is indisputable that tuberculosis is a complex disease with a strong genetic link.

## Data Availability Statement

The datasets presented in this study can be found in online repositories. The names of the repository/repositories and accession number(s) can be found in the article/**Supplementary Material**.

## Ethics Statement

The studies involving human participants were reviewed and approved by The Human Research Ethics Committee of the Federal University of Amazonas (N°. CAAE: 57978916.3.0000.5020, August 17th, 2016). The patients/participants provided their written informed consent to participate in this study.

## Author Contributions

MBAF: realized the experiments, analyzed the data and wrote the paper. DL: analyzed the data and intellectual contribution. AB: analyzed the data and intellectual contribution. MGNF: Sample processing and intellectual contribution. IA: Contributed with samples and patient selection and intellectual contribution. JM: Contributed with samples and patient selection and intellectual contribution and intellectual contribution. TR: Sample processing and intellectual contribution. LF: Sample processing and intellectual contribution. AG: Sample processing and intellectual contribution. AC: intellectual contribution. RR: wrote the paper and intellectual contribution. AP: analyzed the data and intellectual contribution. MO: Sample processing, understanding of genotyping assays and intellectual contribution. AS: Analyzed the data, wrote the paper and intellectual contribution. All authors contributed to the article and approved the submitted version.

## Funding

This work was supported by FAPEAM (Edital PPSUS, Processo 062.00663/2014).

## Conflict of Interest

The authors declare that the research was conducted in the absence of any commercial or financial relationships that could be construed as a potential conflict of interest.
