# Supplementary material for: Single-Nucleotide Variants in the AIM2 – Absent in Melanoma 2 Gene (rs1103577) Associated With Protection for Tuberculosis
Source: Front Immunol. 2021 Apr 1;12:604975. doi: 10.3389/fimmu.2021.604975 (PMC8047195; doi:10.3389/fimmu.2021.604975)
Supplement: Supplementary file 1 [file DataSheet_1.docx]

Supplementary Material

**Supplementary Table 1.** The probes used in the study

| **Gene** | **Identification variant** | **Location on chromosome** | **Probe sequence 5’-[VIC/FAM]-3’** |
| --- | --- | --- | --- |
| *AIM2* | rs1103577 | 1:159130525 | TAAAAGGCACTTCCATTCACTCATT**[C/T]**GCTCAAGCCAGAAAGCCGGGAATCA |
| *CARD8* | rs2009373 | 19:48216157 | TGCTATCAAAAAAAAAAAAGCATGA**[C/T]**TGTGACACCTGGAAAAAATCTTTTT |
| *CTSB* | rs1692816 | 8:11846948 | ACACAGCCCTCTTCCCCAGCCCCTC**[A/C]**CCTGCCTGCCCAATCCAGCCCTATT |

**Supplementary Table 2.** Results of SNVs association analysis on genes coding for AIM2, CARD8 and CTSB inflammasomes in patients with pulmonary tuberculosis (PTB), extrapulmonary tuberculosis (EPTB) and healthy control subjects.

|  | | | | | **Pulmonary tuberculosis vs Control** | | | | **Extrapulmonary tuberculosis vs Control** | | | | **Pulmonary vs extrapulmonary tuberculosis** | | | |
| --- | --- | --- | --- | --- | --- | --- | --- | --- | --- | --- | --- | --- | --- | --- | --- | --- |
| Gene  ID SNV | Genotypes  n, % | PTB  n, % | EPTB  n, % | Controls  n, % | *p* value  (model) | OR  (95% CI) | *p* adj  (model) | OR adj  (95% CI) | *p* value  (model) | OR  (95% CI) | *p* adj  (model) | OR adj  (95% CI) | *p* value  (model) | OR  (95% CI) | *p* adj  (model) | OR adj  (95% CI) |
| *AIM2*  rs1103577 | CC  CT  TT | n=(385)  124 (0.32)  180 (0.47)  81 (0.21) | n=(118)  33 (0.28)  53 (0.45)  32 (0.27) | n=(351)  95 (0.27)  180 (0.51)  76 (0.22) | 0.127  (d) | 0.78  (0.57-1.07) | **0.027**  **(d)** | 0.69  (0.50-0.96) | 0.228  (r) | 1.35  (0.83-2.17) | 0.237  (r) | 1.34  (0.83-2.17) | 0.172  (r) | 1.40  (0.87-2.24) | 0.203  (r) | 1.37  (0.85-2.21) |
| *CARD8*  rs2009373 | CC  CT  TT | n=(376)  111 (0.30)  183 (0.49)  82 (0.22) | n=(92)  31 (0.33)  50 (0.55)  11 (0.12) | n=(353)  109 (0.31)  173 (0.49)  71 (0.20) | 0.574  (r) | 1.11  (0.77-1.58) | 0.420  (r) | 1.16  (0.81-1.68) | 0.061  (r) | 0.54  (0.27-1.07) | 0.064  (r) | 0.54  (0.27-1.07) | **0.026**  **(r)** | 0.49  (0.25-0.96) | **0.026**  **(r)** | 0.48  (0.25-0.96) |
| *CTSB*  rs1692816 | AA  AC  CC | n=(385)  103 (0.27)  193 (0.50)  89 (0.23) | n=(51)  19 (0.37)  17 (0.33)  15 (0.29) | n=(351)  93 (0.27)  169 (0.48)  89 (0.25) | 0.478  (r) | 0.89  (0.63-1.24) | 0.323  (r) | 0.84  (0.59-1.19) | **0.045**  **(o)** | 0.54  (0.29-1.00) | **0.046**  **(o)** | 0.54  (0.29-1.00) | **0.022**  **(o)** | 0.50  (0.27-0.92) | **0.026**  **(o)** | 0.50  (0.27-0.94) |

Legend: ID SNV: Single-nucleotide variant Polymorphism Identification number; genotypes and alleles of frequencies for pulmonary tuberculosis (PTB), extrapulmonary tuberculosis (EPTB) and healthy control (Controls), *p* value (model), odds ratio (OR) and 95% confidence interval (CI). Adjusted for sex and age (padj, ORadj). The best genetic models (dominant: d; recessive: r; overdominant: o) are reported.

**Supplementary Figure 1.** IL-1β profile from pulmonary tuberculosis patients and control and graphic Normal QQ plot for three SNV studied.

Legend: Groups were further compared with each other using two-tailed unpaired Student’s t-test, *** *p*=0,0003.
